# Adjunct use of mouth rinses with a sonic toothbrush accelerates the detachment of a *Streptococcus mutans* biofilm: an in vitro study

**DOI:** 10.1186/s12903-020-01144-0

**Published:** 2020-06-03

**Authors:** Tatsuya Ohsumi, Shoji Takenaka, Yuuki Sakaue, Yuki Suzuki, Ryoko Nagata, Taisuke Hasegawa, Hayato Ohshima, Yutaka Terao, Yuichiro Noiri

**Affiliations:** 1 Division of Cariology, Operative Dentistry and Endodontics, Department of Oral Health Science, Niigata University Graduate School of Medical and Dental Sciences, 5274, Gakkocho-dori 2-Bancho, Chuo-ku, Niigata, 951-8514 Japan; 2 Division of Anatomy and Cell Biology of the Hard Tissue, Department of Tissue Regeneration and Reconstruction, Niigata University Graduate School of Medical and Dental Sciences, Niigata, Japan; 3 Division of Microbiology and Infectious Diseases, Department of Oral Health Science, Niigata University Graduate School of Medical and Dental Sciences, Niigata, Japan

**Keywords:** Biofilm, *Streptococcus mutans*, Mouth rinse, Detachment, Sonic toothbrush

## Abstract

**Background:**

The aim of this in vitro study was to examine the possible enhancement of the biofilm peeling effect of a sonic toothbrush following the use of an antimicrobial mouth rinse.

**Methods:**

The biofilm at a noncontact site in the interdental area was treated by sound wave convection with the test solution or by immersion in the solution. The biofilm peeling effect was evaluated by determining the bacterial counts and performing morphological observations. A *Streptococcus mutans* biofilm was allowed to develop on composite resin discs by cultivation with stirring at 50 rpm for 72 h. The specimens were then placed in recesses located between plastic teeth and divided into an immersion group and a combination group. The immersion group was treated with phosphate buffer, chlorhexidine digluconate Peridex™ (CHX) mouth rinse or Listerine® Fresh Mint (EO) mouth rinse. The combination group was treated with CHX or EO and a sonic toothbrush.

**Results:**

The biofilm thickness was reduced by approximately one-half compared with the control group. The combination treatment produced a 1 log reduction in the number of bacteria compared to the EO immersion treatment. No significant difference was observed in the biofilm peeling effect of the immersion group compared to the control group.

**Conclusions:**

The combined use of a sonic toothbrush and a mouth rinse enhanced the peeling of the biofilm that proliferates in places that are difficult to reach using mechanical stress.

## Background

Dental biofilm control is the most important method of preventing dental caries and periodontal disease. Toothbrushing plays a central role in self-care. Sonic toothbrushes have been reported to remove dental biofilm better than manual toothbrushes [1–3]. Sonic toothbrushes have been developed to improve and promote oral hygiene [4], and have been widely promoted as a tool that easily and effectively removes dental biofilm [4]. In addition, electric toothbrush is beneficial in maintaining the oral health of patients with neuromuscular disorders and reducing the burden of caregivers in completing oral care. Electric toothbrushes have been shown to be an effective adjunct method for plaque control, particularly in patients with a low dexterity level [5]. Auxiliary cleaning instruments must be used to control dental biofilm in the areas where the sonic toothbrush is unable to directly contact the dental surface (e.g., the interproximal area), even though sonic vibrations. The presence of a residual dental biofilm in the interdental areas remains a problem [6, 7].

Various methods have been used to clean the interdental area, such as toothpicks, dental floss, and interdental brushes. Among these options, the interdental toothbrush is considered the most effective method [8, 9]. However, some people experience difficulty using auxiliary cleaning tools, such as interdental toothbrushes, particularly in between the molars. Noncompliance with interdental cleaning is a key issue in self-care, as inadequate compliance leads to periodontal disease [10, 11]. Generally, clinicians postulate that self-care will be more effective if the interdental biofilm is removed with a sonic toothbrush. Dental biofilm removal by sonic toothbrushes at non-contact sites has been evaluated in some studies [12–14]. Even when a sonic toothbrush is used like a manual toothbrush, the complete removal of the dental biofilm in noncontact areas is difficult to achieve.

A previous study investigated the effect of the shear stress of the water current generated by a sonic toothbrush on dental biofilm removal [12]. According to that study, high speed bristle motion creates turbulence in the oral cavity that produces shear stress parallel to the tooth surface. The shear stress may remove the dental biofilm in areas not in direct dental contact with the brush [15, 16].

According to another study, dental biofilm removal at interproximal sites that are not in direct contact with the sonic vibrations from the toothbrush is limited [17]. A mouth rinse represents a chemical control method that can be used as an auxiliary tool to address this deficiency. As shown in our previous study, penetration and disinfection are obtained with mouth rinse alone, but the biofilm structure is not completely removed [18]. *Streptococcus mutans* produces acids from sugar metabolism and uses sucrose to synthesize extracellular polysaccharides (EPS). EPS is a glycan primarily involved in the development and protection of dental biofilms. Antimicrobial compounds have been shown to not function as intended [19]. This phenomenon can be explained by the degradation or delayed penetration by extracellular macromolecules (EPS) in biofilms. In other words, the biofilm cannot be removed only by immersion in the mouthwash. In one study, there was a report that the effect of chlorhexidine digluconate on biofilms caused a concentration gradient in the antimicrobial component, which was below the minimum inhibitory concentration and enhanced biofilm formation [20]. When a dental biofilm is disinfected with a mouth rinse, its structure remains at the dental adhesion interface and may promote new bacterial adhesion [21]. The combination of the mechanical stress from the toothbrush and an antimicrobial mouth rinse exerts a synergistic effect on biofilm dispersal. This in vitro study examined whether an antimicrobial mouth rinse enhances the biofilm peeling effect of a sonic toothbrush. Namely, the sonic brush was used when a small amount of the mouthwash was present in the mouth, or after the application of the mouthwash that filled the interdental portion. An interdental *Streptococcus mutans* biofilm model was used to evaluate the removal of a dental biofilm by a sonic toothbrush in places where the toothbrush does engage in direct dental contact.

## Methods

### Saliva collection and processing

A sterilized saliva solution was prepared using a previously described method [22]. Unstimulated saliva was collected from a healthy person (one of the authors) who had not eaten, drank, or brushed for at least 2 h prior to saliva collection. Saliva samples were diluted (1:10) with sterilized Ringer’s solution containing 0.05% cysteine (Sigma-Aldrich, St. Louis, MO). Diluted solutions were centrifuged at 2000 g for 10 min to remove any debris, and the supernatants were filter sterilized. The saliva solution was used to coat discs with salivary pellicles [18, 23]. All study protocols were approved by the Ethics Committee for Clinical Research of Niigata University (approval no. 2019–0002).

### Preparation of the biofilm structure

*S. mutans* ATCC 25175, which was originally isolated from carious dentin, was purchased from the American Type Culture Collection and was cultured anaerobically at 37 °C on brain heart infusion (BHI) (Difco Laboratories, Detroit, MI) agar plates. Single colonies were selected, inoculated in BHI broth without sucrose, and incubated overnight at 37 °C under anaerobic conditions. The preculture was transferred to 10 ml of fresh BHI broth containing 0.5% sucrose under anaerobic conditions and cultured for 4 h at 37 °C under aerobic conditions. The absorbance of all bacterial suspensions at 600 nm was adjusted to 0.05 prior to inoculation.

**Fig. 1:**
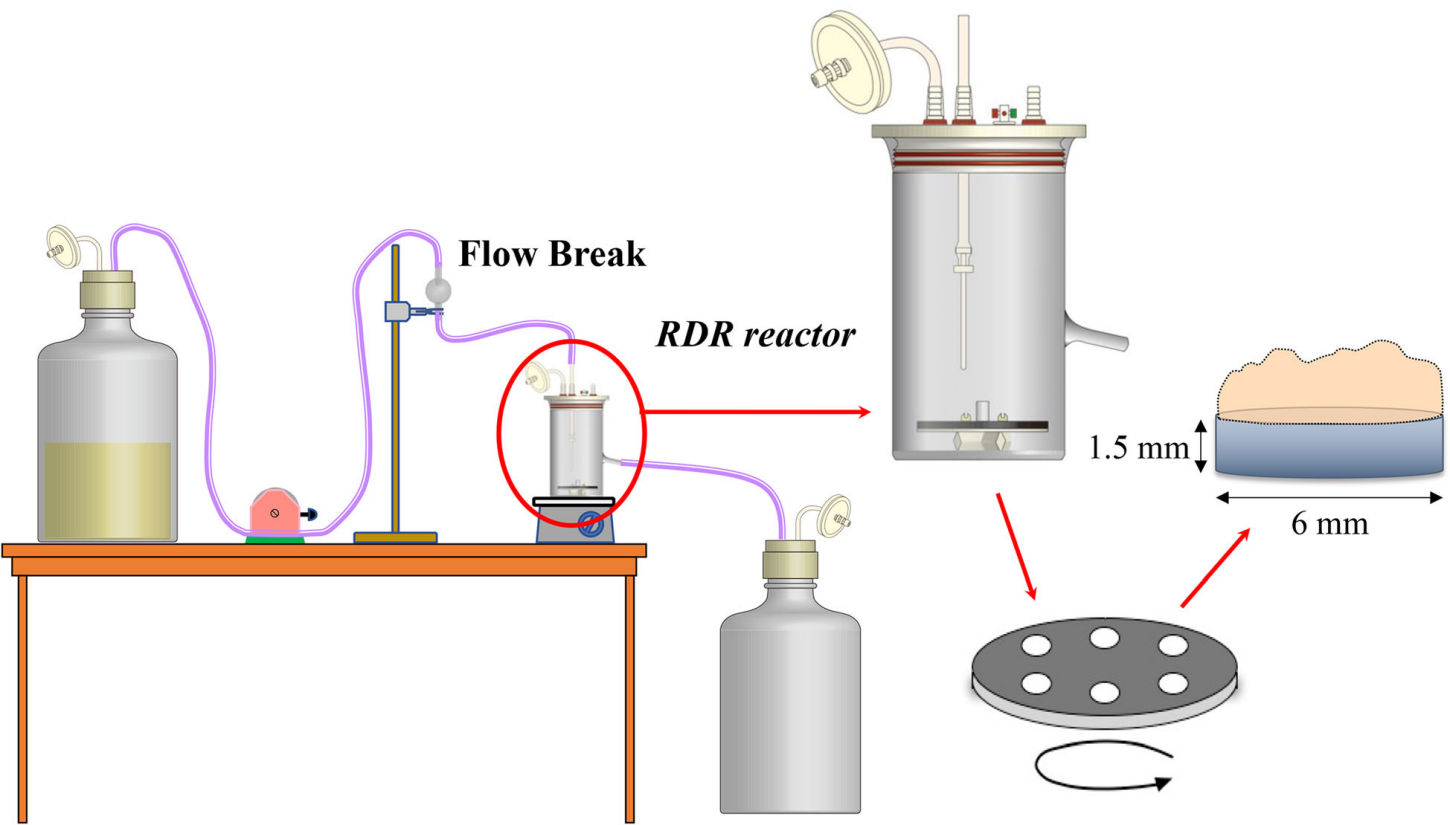
Schematic of the rotating-disc biofilm reactor system used to grow the *Streptococcus mutans* biofilms. The rotating-disc biofilm reactor is shown in the continuous flow mode of operation. The reactor was used to develop a standardized three-day *S. mutans* (ATCC 25175) biofilm on resin composite discs (Premise Flowable, Kerr). The size of the resin composite disc was standardized to 6 mm in diameter and 1.5 mm in thickness. The rotor was composed of a magnetic stir bar, on which a rotating-disc was attached to hold resin composite discs. The container equipped with the stirring bar was placed on a magnetic stirrer and incubated while rotating. Disc rotation provided continuous mixing of the solution added to the system

Composite resin materials (Premise Flowable, Kerr, Orange, CA) were used as the attachment site for the biofilm structure. Standardized discs, 6 mm in diameter and 1.5 mm in thickness, were prepared and polished with 4000 grit waterproof silicon carbide paper; then, they were subjected to ethylene oxide gas sterilization for 4 h. The discs were coated with 10% conditioned sterile saliva for 2 h at room temperature. Biofilm structures were prepared as previously described [21]. *S. mutans* biofilms were allowed to form on the discs using a rotating-disc reactor (RDR) (Biosurface Technologies Corp., Bozeman, MT). This system is depicted in Fig. 1 and has been previously described in detail [24].

The discs were incubated for 90 min at 37 °C in the BHI broth without sucrose, containing the *S. mutans* cell suspension while stirring at 75 rpm to achieve initial adhesion. Following the adhesion phase, the stir disc was gently rinsed with 100 ml of phosphate buffer (5.0 g l^− 1^ NaCl and 2.5 g l^− 1^ Na_2_HPO_4_, pH 7.4), and was aseptically transferred to a sterile reactor vessel filled with 300 ml of diluted BHI broth (1:10) containing 0.05% sucrose. The biofilm was allowed to form for 72 h while the solution was stirred at 50 rpm under continuous flow aerobic conditions at a rate of 4.6 ml min^− 1^ during an incubation at 37 °C. The medium was changed every 12 h. After the fixed incubation period, the rotating wheel was aseptically removed, and the specimens were washed three times with phosphate buffer.

### Treatment

**Fig. 2:**
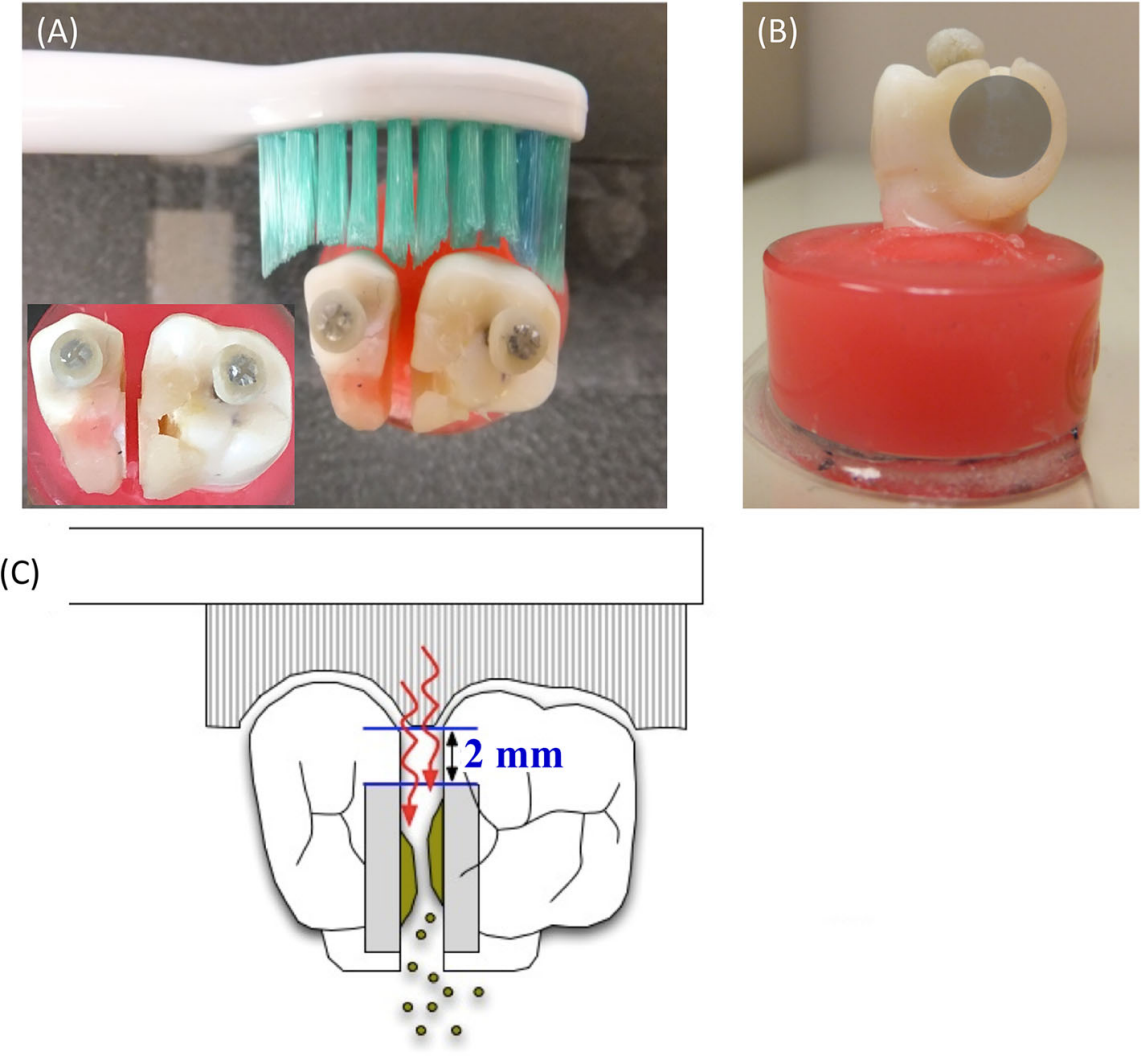
Interdental model of plastic teeth with mounted discs. The resin composite discs are separated from the reach of the toothbrush bristles by 2 mm. The plastic teeth are positioned such that brushing is applied to the buccal surface (**a**). Inset: The discs are held perpendicular to the plane of the brushing action, the gap mimicking the embrasure. The approximate position of the toothbrush is shown in (**b**). Oral devices are positioned perpendicularly to the fixed specimens, as illustrated in (**c**) for the sonic toothbrush. A gap was provided to ensure that the tip of the toothbrush did not hit the tooth, and the position of the electric toothbrush was set

A pair of resin composite discs containing biofilms was inserted into recesses located between plastic teeth (Fig. 2a). These plastic teeth were then placed into a typodont model (Nissin Dental Products, Inc., Kyoto, Japan) located inside an exposure chamber containing either phosphate buffer or mouth rinses. The specimens were divided into six groups. Two independent experiments were performed to obtain *n* = 7 specimens per group. Each experiment consisted of an immersion group and a combination group. The immersion groups were treated with phosphate buffer (group C), a chlorhexidine digluconate Peridex™ (CHX) alcohol-containing (11.6%) mouth rinse (3 M ESPE, USA) (group G), or a Listerine® Fresh Mint (EO) alcohol-containing (21.6%) mouth rinse (Johnson & Johnson, USA) (group L). The combination groups received the test mouth rinses and treatment with a sonic toothbrush (designated in the groups as ST) (Philips Sonicare Flexcare HX6930; ST). The combination groups were designated as the C + ST group, the G + ST group, and the L + ST group.

In the immersion groups, the biofilm constructs were immersed in the tested solution for 5 s. In the ST combined groups, the biofilms were treated with a sonic toothbrush for 5 s at a position located 2 mm away from the disc (Fig. 2b, c). Thereafter, the discs with biofilms were gently rinsed with sterile phosphate buffer three times to remove excess treatments, and the residual biofilm structure was observed with a scanning electron microscope (SEM). In addition, the biofilm was recovered by an ultrasonic treatment, the viable cell count was estimated using the colony counting method, and the total number of bacteria was calculated using the PCR-Invader method.

### SEM imaging

After treatment, the biofilm structure was observed with a SEM (EPMA-1610, Shimadzu, Kyoto, Japan). Specimens were washed with phosphate buffer and fixed with 2.5% glutaraldehyde for 2 h. After fixation, the fixed specimens were dehydrated using a series of ethanol solutions (10 min each in 60, 70, 80, 90, 95, and 100% ethanol) and then air dried. The samples were sputtered with gold-palladium and examined using the SEM.

### Cryo-embedding, cryo-sectioning, and measurement of the thickness of the *S. mutans* biofilms

The treated biofilm samples were embedded in a protective medium (Tissue-Tek O.C.T. compound, Sakura Finetek, Tokyo, Japan) as previously described [25]. The resin discs were placed on dry ice, and the medium was gently poured on them from above and allowed to freeze. The resin discs were peeled away from the embedded medium to ensure that the biofilm remained attached to the medium side. Then, the biofilm was placed on dry ice with the embedded side down. The embedding medium was used to cover the bottom surface of the exposed biofilm. The embedded biofilm samples were sectioned into 8 μm cross sections using a cryostat (CM 3050 S; Leica, Nussloch, Germany). The thickness of the biofilm was measured and partitioned into 10 section intervals. Twenty-five sections from each embedded sample were analyzed.

### Quantitative analysis of viable and total cells

Colony counts and the PCR-Invader assay were used to quantify the numbers of viable and total bacteria in the test samples, respectively. Samples were washed three times with phosphate buffer and immersed in 3 ml of phosphate buffer. Biofilms were collected by vortex mixing for 3 min, followed by ultrasonication for 5 min. Samples were serially diluted in autoclaved distilled water, and 100 ml of each dilution were plated on BHI agar. The plates were incubated anaerobically for 48 h at 37 °C, after which the number of viable colonies were counted.

The total number of bacteria was determined using the modified Invader PLUS method developed by BML Inc. (Saitama, Japan). The details of the PCR-Invader assay have been previously reported [26]. The PCR-Invader assay combines polymerase chain reaction amplification and invader detection, and can calculate the total number of bacteria with high sensitivity and speed. The primers for *S. mutans* were based on a region of the 16S ribosomal RNA sequences. Bacterial DNA was extracted using Pure LC (Roche, Tokyo, Japan) and a MagNA Pure LC Total Nucleic Acid Isolation Kit (Roche). The template DNA (3 ml) was added to 12 ml of a reaction mixture containing 20 mM primers, 2.5 mM dNTP, 2.5 U of AmpliTaq gold, 3.5 mM primary probe, 0.35 mM Invader oligo, and the Invader core reagent kit, which consisted of FRST mix and enzyme/MgCl_2_ solution (F-primer, 5′-GGATTCGCTAGTAATCG-3′; R-primer, 5′-TACCTTGTTACGACTT-3′; Tb-Primary probe, 5′-CGCGCCGAGGCCGGGAACGTATTCACC-3′; and Tb-Invader oligo, 5′-TGACGGGCGGTGTGTACAAGGCA-3′). Reaction mixtures were preheated at 95 °C for 20 min, and then a two-step PCR was performed for 35 cycles (95 °C for 1 s and 63 °C for 1 min) using the ABI PRISM 7900 sequence detection system (Applied Biosystems, Foster City, CA). Fluorescence values for carboxyfluorescein (wavelength/bandwidth: excitation 485/20 nm; emission 530/25 nm) were measured at the end of the incubation/extension step at 63 °C for each cycle. Each assay was performed in triplicate and the mean values from the six independent samples were determined.

### Statistical analysis

Statistical analyses were performed using the SPSS 11.0 (IBM, Armonk, NY) program. When applicable, data are presented as means ± standard deviations (SD). The significance of measurement biofilm thickness, viable and total cell counts were determined using Kruskal-Wallis test and the post hoc Steel-Dwass test.

## Results

### SEM observations

**Fig. 3:**
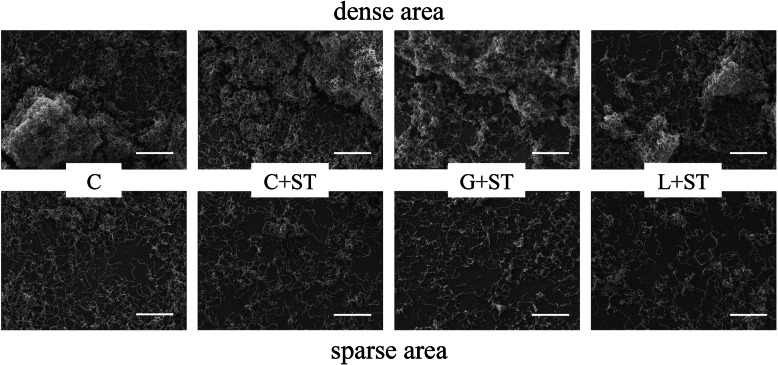
SEM images of the biofilm structure remaining on the disc after each experimental treatment (C, C + ST, G + ST, and L + ST). The upper row depicts representative SEM images of the dense area of the biofilm structure in each experimental group. The lower row shows representative SEM images of the sparse area of the biofilm structure in each experimental group. Scale bar, 20 μm

SEM images of high and low cell density areas were captured from each group (Fig. 3). No apparent difference in the cell density areas was observed between group C and the C + ST and the G + ST groups. On the other hand, the biofilm structure in the cell density area remaining on the disc tended to be reduced in the L + ST group. A decrease in the number of bacteria was observed in the L + ST group after sonic toothbrush treatment in areas with high and low cell density compared to the other groups. In the sparse areas, the biofilm exhibited a monolayer structure, and the intercellular density tended to decrease in the following group order: C, C + ST, G + ST, and L + ST (Fig. 3, lower row).

### Biofilm thickness

**Fig. 4:**
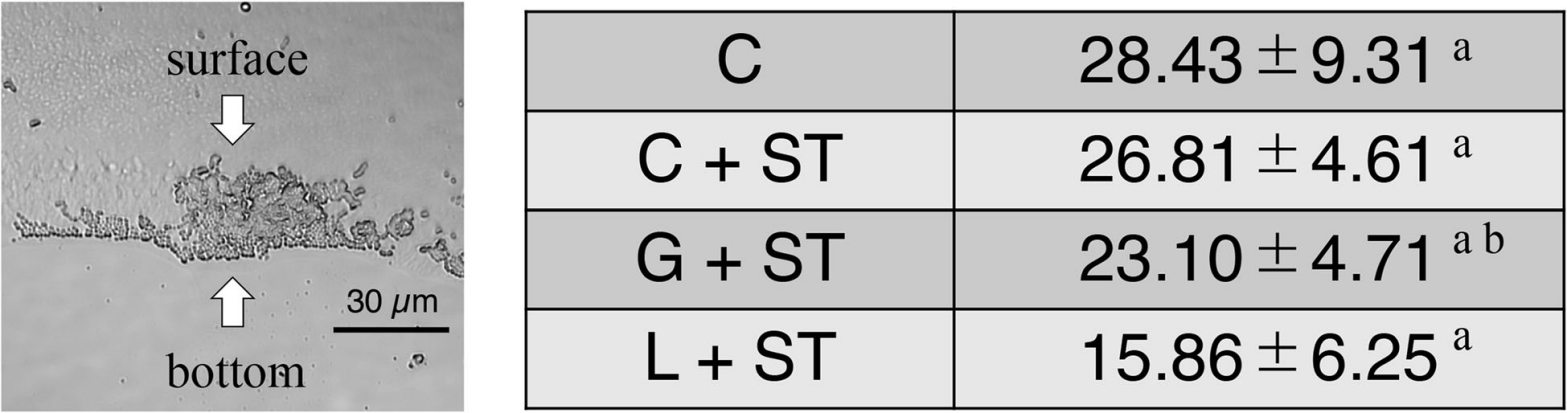
Comparison of the thickness of the biofilm structure between the control group (C) and the sonic toothbrush combined groups (C + ST, G + ST, and L + ST). The left panel shows a representative transmission image of a frozen longitudinal section of the biofilm (scale bar, 30 μm). The biofilm thickness was measured from the bottom surface of the biofilm to the top surface of the biofilm. The thickest parts were measured for each section. Data are presented as means ± SD (*n* = 25, Steel-Dwass test). Same alphabetic character indicates that values are not significantly different (*p* > 0.05)

Using the frozen longitudinal sections, the biofilm height after the experimental treatments was measured. The maximum thickness of the residual biofilm structure that developed in this study was approximately 30 μm for the control. The images of cryo-sections (Fig. 4) provided a higher resolution of the deeper layer. The thickness of the L + ST group was significantly less than the control groups and the C + ST groups (*p* < 0.05 for the L + ST group).

### Quantitative analysis of the numbers of viable and total cells

**Fig. 5:**
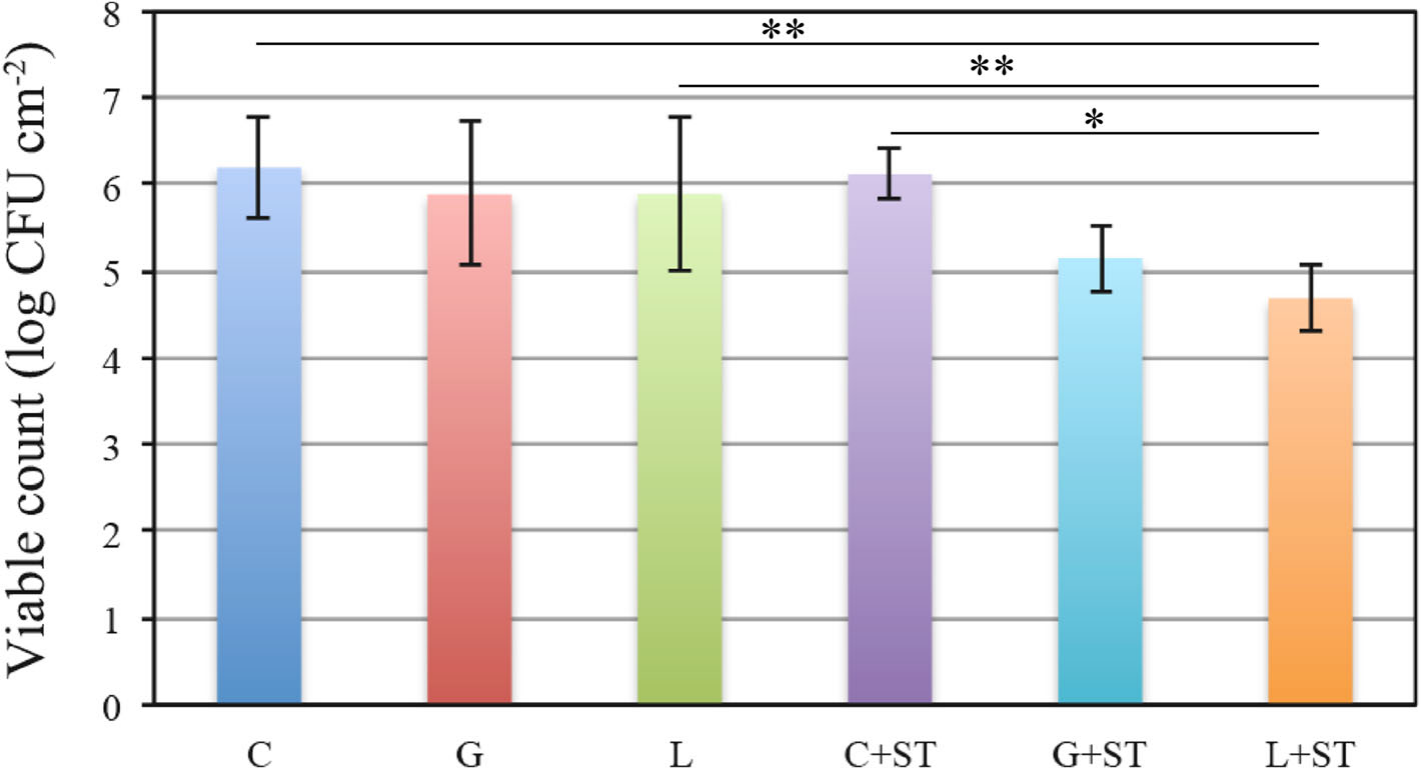
Calculation of the number of live cells remaining on the resin discs after treatment for 5 s. Colonies were counted and the bacterial load was calculated. The bacterial load was assessed as the number of bacteria in colony forming units (CFU) per cm^− 2^ (corresponding to the unit area of one resin disc). Data are presented as means ± SD (*n* = 7, one-way ANOVA, Steel-Dwass test *p < 0.05 and **p < 0.01). Error bars represent 95% confidence intervals

**Fig. 6:**
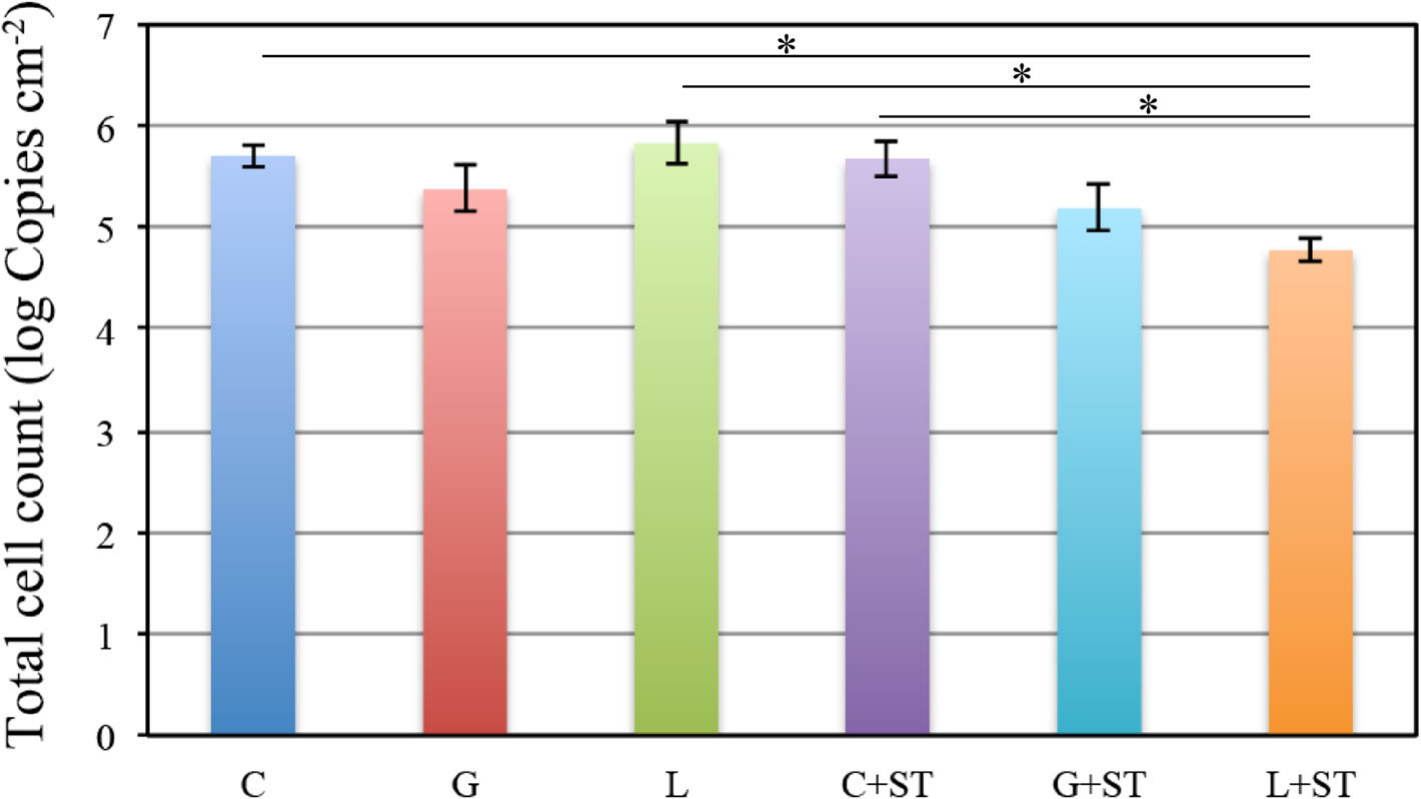
Total number of *S. mutans* cells present in the biofilm remaining on the resin discs after an immersion and a combination treatment for 5 s. The total bacterial load was assessed as the number of bacteria determined using the PCR-invader assay per cm^− 2^ (corresponding to the unit area of one resin disc). Data are presented as means ± SD (*n* = 7, one-way ANOVA, Steel-Dwass test *p < 0.05). Error bars represent 95% confidence intervals

The number of viable cells (Log CFU ± SD/ml) was 6.20 ± 0.59 (Group C), 5.89 ± 0.83 (Group G), 5.89 ± 0.89 (Group L), 6.12 ± 0.30 (C + ST Group), 5.15 ± 0.39 (G + ST Group), and 4.69 ± 0.38 (L + ST Group). A significant reduction in the number of viable cells was observed in the L + ST group compared with all the other groups (*p* < 0.01 for the C and L groups; p < 0.05 for the C + ST group). A significant difference was not observed between the C, L and C + ST groups (*p* > 0.05) (Fig. 5). The total number of bacteria (Log CFU ± SD/ml) was 5.72 ± 0.11 (Group C), 5.38 ± 0.23 (Group G), 5.97 ± 0.19 (Group L), 5.68 ± 0.18 (C + ST Group), 5.20 ± 0.24 (G + ST group), and 4.79 ± 0.11 (L + ST group). The total number of bacteria was significantly reduced in the L + ST group (p < 0.05) compared with all the other groups. A significant difference was not observed between the C, L, and C + ST groups (p > 0.05) (Fig. 6).

## Discussion

A quantitative analysis of the viability of bacteria present in dental biofilms was performed after various treatments with a mouth rinse and a sonic toothbrush. Hydrodynamic phenomena and microbiological aspects of the biofilm were also studied.

After the usual brushing, a mouth rinse was used as an adjunct. The water flow generated by the sonic toothbrush was carefully monitored to increase the effectiveness of the mouth rinse. The sonic toothbrush produces a liquid flow to the interdental area, and a fluid shear stress removes the biofilm [13]. Therefore, we hypothesized that the fluid shear stress, mouth rinse penetration, and bactericidal effect would combine to produce a synergistic effect when a mouth rinse containing an antimicrobial component was used. Although the biofilm was not eliminated from the dental surface, our results conform to the general consensus that the sonic toothbrush, namely, a toothbrush with bilateral symmetry and multidimensional and sonic action, enables biofilm removal in non-contact areas [27, 28]. According to the literature, a more pronounced reduction in the biofilm is observed after the use of a side-to-side toothbrush than after the use of a toothbrush with multidimensional action [29–32]. However, the application of these findings to a type of electric toothbrush that was not analyzed in this study should be avoided. In addition, since the present study focused on biofilm exfoliation, the altered distribution of viable and dead biofilm-forming bacteria after non-contact brushing treatment was not verified.

A sonic toothbrush generates microbubbles in the surrounding liquid, which is called cavitation. These bubbles contact the biofilm, creating shear stress that results in peeling of the dental biofilm [13], namely, removing the biofilm from areas that are not receiving direct contact from the bristles.

The mechanism by which the biofilm is peeled off is through shear stress. Some biofilms that have grown to a certain height exhibit physical smoothness due to saliva, food, and other materials that diffuse out from the surface layer. Shear stress does not remove biofilm from the interdental areas, allowing the biofilm to accumulate. An in vitro model in which a dental biofilm was deposited in the interdental areas was constructed to mimic this effect.

As mentioned above, sonic toothbrushes generate bubbles in connection with increased liquid flow. Shear stress is exerted by these bubbles passing through the interdental areas [29]. We hypothesized that the combination of the sonic vibration of the sonic toothbrush with the antibacterial action of the mouth rinse would improve the removal of the dental biofilm removal compared to the use of the toothbrush alone. Synergistic effects were expected for the use of chemical mouth rinses in combination with mechanical toothbrushing.

As a method for further enhancing the biofilm removal effect of a sonic toothbrush and mouth rinse, a surface coating agent is applied to suppress the adhesion of bacteria to the teeth. By suppressing adhesion or reducing the adhesion capability, mechanical removal is facilitated. This coating would allow the shear stress of the water stream from the sonic toothbrush to more easily peel off any attached biofilm [33].

In the present artificial biofilm model, even when using EO, CHX, and the sonic toothbrush, significant biofilm peeling was not achieved with only 5 s of toothbrush action. *S. mutans* adheres firmly to the interface by producing sticky glucans [34, 35]. The shear stress generated by the water flow created by the sonic brush exerts a peeling effect on the biofilm attached to the interface [28]. No significant difference was observed. However, in the presence of CHX or EO mouthwash, the release effect was significantly improved. In addition, no significant difference was observed in the bactericidal effect of antibacterial components in the mouthwash. This lack of a difference is thought to be due to the fact that glucan, a constituent of the biofilm of *S. mutans*, impedes the penetration of antibacterial components and diminished the effect. We surmised that the bactericidal action was not substantially affected because of the short application time of 5 s. However, the biofilm peeling effect on the adjacent surfaces where the biofilm is difficult to mechanically remove was enhanced using the combination of the mouth rinse and the sonic toothbrush. Given the daily use of sonic toothbrushes, the application of the toothbrush to the same place for long periods is not realistic. Although 5 s is a relatively short action time, the peeling of the biofilm was significantly enhanced when the sonic toothbrush was used in combination with the mouth rinse. A comparison of the two mouth rinses revealed that EO exerted a greater peeling effect than chlorhexidine. A potential explanation for this finding is a difference in the penetration rate of the antimicrobial component of the mouth rinse [36]. In the report by Wakamatsu et al., the permeation rate of the antimicrobial component of EO was significantly higher in a 30 s comparison. EO penetration was faster than CHX, but shows how difficult it is for antibiotics to penetrate oral biofilms for a short time [18]. This result was similar to the result of this experiment because no difference was observed in CFU of the group that was only immersed. In contrast, the peeling action of the mouth rinse combined with a sonic brush is presumed to be mediated by a weakening of the binding force of the biofilm matrix by the mouthwash.

As countermeasures against oral biofilms, chemical control methods using antibacterial ingredients are applied in anticipation of enhancing the mechanical effect of toothbrushing. Due to the cavitation effect, the dental biofilm outside the range of the sonic toothbrush bristles may also be removed by the water current, if the biofilm adhesion is weak. The treatment and prevention of periodontal disease requires daily dental biofilm removal. Failure to comply with oral hygiene measures is frequently observed, particularly in difficult to reach areas, such as interdental areas [37, 38]. If the sonic toothbrush effectively removes dental biofilm without making bristle contact, oral hygiene will improve. However, evidence supporting this hypothesis is currently unavailable in clinical settings. After comparing the in vitro data from the present study, the effects of brushing protocols on biofilm formation must be considered.

The difference between an oral biofilm and the *S. mutans* biofilm model used in this study should be taken into account. Clinical phenomena are not necessarily reproduced using a single bacterial strain. Although *S. mutans* is not representative of the complex group of microorganisms typically detected in dental biofilm, *S. mutans* easily forms biofilms and has been used as a model in various biofilm studies [39, 40]. The glucan-containing extracellular polysaccharide produced by *S. mutans* strengthens the biofilm, increasing the difficulty of removal by brushing. Therefore, in future studies, we plan to combine the application of the sonic toothbrush with an enzyme that decomposes extracellular polysaccharides.

## Conclusion

EO and CHX mouth rinses were used in combination with a sonic toothbrush to evaluate dental biofilm removal from an in vitro model. The combined effect of the sonic toothbrush and the mouth rinse enhanced biofilm peeling from the adjacent dental surfaces that the toothbrush is unable to reach directly. Individual deficiencies in the chemical and mechanical removal methods are addressed by the synergistic combination.

## Data Availability

The datasets used and analyzed during the current study are available from the corresponding author on reasonable request. The *S. mutans* genome sequence is available through GenBank (accession no. AE014133).

## Abbreviations

EO: Listerine® Fresh Mint
CHX: Chlorhexidine digluconate Peridex™
BHI: Brain heart infusion
RDR: Rotating-disc reactor
group C: Immersion groups were treated with phosphate buffer
group G: Immersion groups were treated with a CHX alcohol-containing (11.6%) mouth rinse
group L: Immersion groups were treated with an EO alcohol-containing (21.6%) mouth rinse
ST: Sonic toothbrush
SEM: Scanning electron microscope
SD: Standard deviations
